# A subtype of oral, laryngeal, esophageal, and lung, squamous cell carcinoma with high levels of TrkB-T1 neurotrophin receptor mRNA

**DOI:** 10.1186/s12885-019-5789-8

**Published:** 2019-06-20

**Authors:** Yalu Zhou, Saurabh Sinha, Joel L. Schwartz, Guy R. Adami

**Affiliations:** 1 Department of Oral Medicine & Diagnostic Sciences, Center for Molecular Biology of Oral Diseases, College of Dentistry, University of Illinois at Chicago, 801 South Paulina Street, Chicago, IL 60612 USA; 2 Department of Computer Science and Carl R. Woese Institute of Genomic Biology, University of Illinois at Urbana-Champaign, 2122 Siebel Center, 201N. Goodwin Ave, Urbana, IL USA

**Keywords:** NTRK2, TRKB-T1, Squamous cell carcinoma, NFE2L2, SOX2, PIK3CA, Sonic hedgehog

## Abstract

**Background:**

The NTRK2 genetic locus encodes neurotrophin membrane receptors that play an important role in normal neural tissue plasticity, growth, and survival. One NTRK2-encoded protein is TrkB-FL, which can regulate multiple pathways relevant to cancer. A second NTRK2 gene mRNA isoform encodes TrkB-T1, a receptor that has a different cytoplasmic domain encoded in a mRNA with a unique 3′ terminal exon.

**Method:**

Tumors from The Cancer Genome Atlas (TCGA) and other studies were classified according to the expression of a single form of NTRK2 mRNA, TrkB-T1, identified by its unique 3′ terminal exon. Analysis of differentially expressed genes in TrkB-T1 high expressers was done to determine if tumors enriched for TrkB-T1 mRNA were a uniform group independent of anatomic site.

**Results:**

The mRNA for TrkB-T1 is the most abundant NTRK2 gene mRNA in all squamous cell carcinomas (SCCs) in the TCGA database. Comparison of larynx SCC high TrkB-T1 RNA expressers to low expressers (*n* = 96) revealed gene expression differences consistent with the high TrkB-T1 tumors being more neural-like. The upregulated genes in the TrkB-T1 RNA high expressers also showed enrichment of pathways involved in retinol metabolism, hedgehog signaling, and the Nfe2l2 response, among other pathways. An examination of oral, esophagus, and lung SCCs (*n* = 284, 97, 501) showed induction of the same pathways among tumors that expressed high levels of TrkB-T1 mRNA. Proteins associated with regulation of the sonic hedgehog pathway, and the Nfe2l2 response, Tp63, and Keap1 and p62/SQSTM1 proteins, showed differential expression in larynx, oral and lung high TrkB1-T1 expresser SCCs. Unexpectantly, the relationship of high level TrkB-T1 expression to patient outcomes was SCC anatomic site specific. High TrkB-T1 mRNA levels in laryngeal SCC correlated with poor survival, but the opposite was true for lung SCC. This may be because pathways enriched in the TrkB high expressers, like those involving oncogenes NFE2L2, PIK3CA, and SOX2, are known to have SCC anatomic site-specific effects on progression.

**Conclusions:**

High level TrkB-T1 mRNA is a marker of a distinct SCC subtype enriched for at least 3 pathways relevant to tumor progression: Nfe2l2 response, retinol metabolism, and hedgehog signaling.

**Electronic supplementary material:**

The online version of this article (10.1186/s12885-019-5789-8) contains supplementary material, which is available to authorized users.

## Background

Squamous cell carcinoma (SCC) is a cancer identified histologically, with the cells resembling, and possibly derived from, the flattened epithelial cells of skin or mucosal epithelium. Nearly 95% of head and neck carcinomas (HNC), 30% of lung cancers, and 50–90% of esophageal cancers, depending on geographic site, are SCCs [1–3]. The tumors, while histologically similar, have distinct treatments depending on the anatomic site. With head and neck SCCs there are at least 4 tumor subtypes based on mRNA gene expression [4, 5] and tumors of the oral pharynx are different in that they often have HPV infection in their etiology [6, 7]. Molecular characterization can be used to classify head and neckl SCCs and has potential in treatment optimization.

The intact, nonmutated TrkB neurotrophin receptor is thought to play a role in progenitor neural cell migration, survival, and differentiation [8–10]. In oral, head and neck, ovarian, pancreatic, colon, prostate, and gastric cancers and neuroblastomas, high TRKB protein levels correlate with worse patient outcomes [10–18]. Rare mutated versions of the NTRK2 gene, which encode proteins consisting of fusions of the TrkB kinase domain with domains of other signaling proteins, are drivers in a number of cancers [17, 19–22]. The intact NTRK2 gene is also a potential oncogene [12, 13, 23]. The largest form of the NTRKB gene products, TrkB-FL, binds Brain Derived Neurotrophic Factor (BDNF) which is known to activate three signaling pathways via autophosphorylation. This is followed by the recruitment of intermediate signaling proteins that activate the PI3K/AKT signaling pathway, which promotes neural cell outgrowth and cell survival, the ERK/MAPK pathways that control survival, and pathways involving phospholipase C that enhance neuronal plasticity [24–27]. While most of the processes have been best studied in nervous system cells, there is good evidence TrkB-FL overexpression can activate PI3K/AKT pathways, MEK/ERK cascades, cell proliferation, epithelial mesenchymal transition, and multiple metastasis-promoting properties in tumors [18, 28]. There is a second form of TrkB, TrkB-T1, also a membrane BDNF receptor, with a distinct C-terminus that lacks its own kinase domain and instead has a unique 12-amino acid at the carboxy terminus of its shortened cytoplasmic domain. TrkB-T1 can form heterodimers with kinase active forms, which may at times work as a dominant negative of TrkB-FL [8, 29]. TrkB-T1 can also compete for binding of BDNF, which it then internalizes [29]. TrkB-T1 plays a major role in neurite filopodia outgrowth in the cell and changes in cytoskeleton in glioma cells [8, 9, 30]. Little else is known mechanistically about how TrkB-T1 alters other cell phenotypes in neural cells, such as proliferation or survival [30–32]. What it does in tumor cells is similarly poorly understood, it has been shown to protect mammary cells from apoptosis in the presence of BDNF and in pancreatic cancer cell lines it can increase proliferation and cell migration [33, 34].

Accurate measurement of NTK2 gene expression can be difficult. The TrkB receptor protein is differentially glycosylated and there are multiple forms produced from differentially spliced NTRK2 gene mRNA [35]. While measurement of TrkB protein levels in tumors may prove useful for prognosis, immunohistochemistry-based quantification is variable among head and neck cancers. The percentage estimates of head and neck SCCS expressing high levels of TrkB vary widely [11, 18, 35–37]. Antibodies that differentiate the most common forms of the protein are not readily available and the molecular weight of the most often studied TrkB-FL is nominally 92KD though it is often identified at 145 KD on denaturing western analysis, a range that overlaps with other isoforms [18, 38]. In many studies it is not clear which form of NTRK2 gene mRNA or protein has been measured [15].

The work here exclusively focuses on the measurement of the two most common forms of NTRK2 gene RNAs: TrkB-FL and TrkB-T1. This work highlights that TrkB-T1 is the most abundant form of the mRNA in all tumor types examined as has been shown for many normal cell types in the body [35]. The ease of reproducible measurement of TrkB-T1 mRNA, via detection of the terminal exon using DNA microarrays or RNASeq, was used to determine if tumors highly enriched for TrkB-T1 mRNA make up a separate subtype of head and neck SCC and to discern pathways that are enriched in this subset of SCCs.

## Methods

The Cancer Genome Atlas (TCGA) gene expression (RNAseq V2), RSEM (RNA-Seq by Expectation-Maximization), CNA copy number amplification and clinical data for the 518 lung adenocarcinoma (LUAD), 501 lung SCC (LUSC), 284 oral SCC (OSCC), 96 laryngeal SCC (LASC), 90 esophageal adenocarcinoma (ESAD), and 97 esophageal SCC (ESSC) samples, data originally curated by TCGA Research Network were obtained from the Broad Institute TCGA GDAC Firehose repository via Firebrowse (http://fbdev/api-down.html). The Cancer Proteome Atlas (TCPA) reverse-phase protein array analysis data (V4.2, 2018/07/18) of 353 LUAD, 322 LUSC, 206 OSCC, and 79 LASC was downloaded from the MD Anderson TCPA website [39] https://www.tcpaportal.org/tcpa/download.html. Level 4 replica-based normalization was used to minimize batch effects.

### Quantification of NTRK2 RNA isoforms

(Calculation of TrkB-T1, TrkB-FL, and TrkB-Shc RNA levels). Exon expression measurement is based on exon quantification file and gene expression is based on normalized gene expression values in RSEM. Gene expression for NTRK2 gene, TrkB-T1 RNA isoform was derived based on the exon 16 quantification, while TrkB-FL RNA was proportional to exons 23 and 24 and TrkB-SHC RNA was proportional to exon 19 [35]. These exons are specific to the different RNAs.

### Identification of DE genes and enriched gene sets

Gene expression datasets were categorized into ‘TrkB-T1 high expressers’ and ‘TrkB-T1 low expressers’ based on TrkB-T1 mRNA levels, with the cutoff at the mean level. DE genes were then identified between high and low TrkB-T1 mRNA expressers, as explained below. For all groups there were substantially more subjects in the low-expresser groups. For esophageal adenocarcinoma (ESAD) this scheme produced less than 15 DE genes, so the top 30 and lowest 30 TrkB-T1 expressers were compared instead to produce a larger DE gene list. All mRNA data were log2 transformed using BRB Array Tools V 4.5 (https://brb.nci.nih.gov/BRB-ArrayTools/index.html), then class comparison was performed using a *t* test with and without permutation of class labels to allow determination of FDR on all RNAs with at least 80% of the values available. Heat map of DE for OSCC was generated for the class comparison including 1140 genes FDR < 0.005.

### Three different methods were used with gene lists from TRK-T1 high expresser tumors to identify enriched biological pathways

The Enrichr tool (http://amp.pharm.mssm.edu/Enrichr/) [40] was applied to the resulting DE gene list to identify significantly overrepresented KEGG pathways (p_adj_ ≤ 0.05).” Enrichr calculates this value by using a corrected Fisher exact test. KnowEnG is a second pathway analysis tool [41]. It uses random walks with restarts to strengthen sensitivity to similarly identify pathways that were enriched, but this time within the Molecular Signature Database, MSigDB, because of its relevance to cancer and its descriptive titles for individual gene sets. The interaction network chosen to aid in identification of relevant genes was STRING Co-expression. For Enrichr and KnowEnG, only DE genes with higher expression of at least 2x and FDR < 0.05 in the high TrkbT1 group were considered, and the top 400 were used. For ESAD, there were 99 DE genes for Gene Set Enrichment Analysis (GSEA) (http://software.broadinstitute.org/gsea/index.jsp), and all were used [42]. The entire expression table was natural log transformed and then entered into the program. All genes were considered in determining gene sets differentially represented in the high and low TrkB-T1 mRNA groups for Larynx + Oral, ESSC, LUSC, and LUAD. A cutoff of FDR q-val > 0.25 was considered significant.

For the comparison to Genotype Tissue Expression (GTEx) Project of normal tissue transcriptomes, Enrichr was used under the category of Cell Types [43].

To determine transcription factors associated with high TrkB-T1 expression, promoter sequence of enriched DE genes were scanned using the position weight matrix method from the TRANSFAC and JASPAR transcription factor binding motifs [44, 45].

### Data visualization

To analyze and visualize mutations, copy number alteration and gene expression across all the samples in the head and neck SCC dataset cBioPortal was used [46, 47]. In that 95% of TrkB mRNA is TrkB-T1 mRNA, for ease of use all data generated via cBioPortal is ranked by TrkB mRNA levels not TrkB-T1 mRNA, which are expected to be near equivalent.

### Statistical tests

Survival curves were generated using Cutoff Finder Web application, though all categorizations of the group pairs were based on separation of the two groups into high expressers and low expressers, with the cutoff at the mean level for TrkB-T1 or other RNA [48]. Logistic regression was used to determine the relation between the presence of CASP8, PIK3CA mutations and NTRK2 expression (Statistical Analysis Software, Cary, NC, USA). All statistical tests were two sided with *P* < 0.05 considered significant. For clinical parameters of high and low TrkB-T1 RNA tumor patients, either Chi Squared or Fisher Exact test when appropriate or in the case of age, the Student *t* test was used to determine statistical significance.

## Results

### Great variability in TrkB-T1 mRNA levels in SCCs

**Fig. 1:**
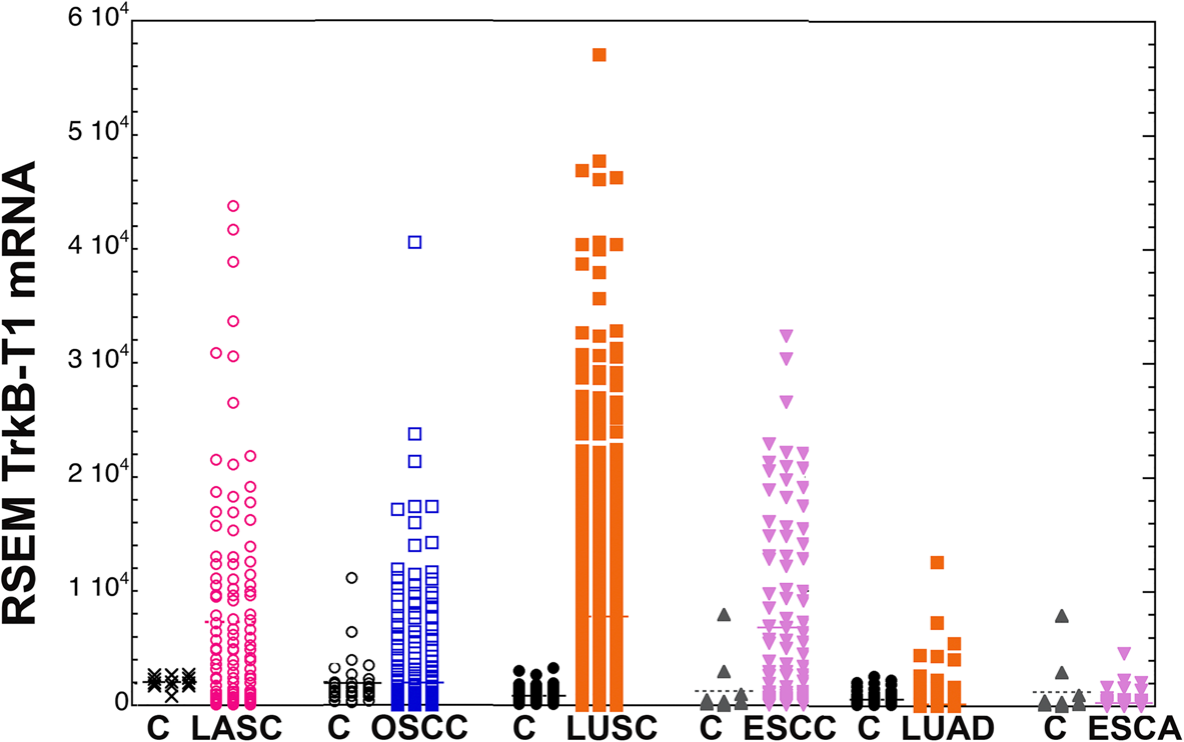
Tumor and corresponding control (C) tissues are compared for expression levels of TrkB-T1 mRNA from the NTRK2 gene for the 6 tumor types. Each object in the tumor or control (C) tri-column represents the TrkB-T1 mRNA level in a single tumor or control site. The means are indicated by the horizontal lines. The y axis for mRNA expression is on a linear scale to accentuate differences

TCGA cohorts of cancer patients provide large datasets of measured mRNA levels in tumors. The mRNA encoding TrkB-T1 contains a unique terminal exon, exon 16, and poly A site providing the encoded protein with a distinct carboxy terminus and serving as a means of identification of this RNA. RNA containing exon 16 comprise over 97% of the RNA coming from the NTRK2 gene in both normal oral mucosa and OSCC. The same was true for laryngeal SCC (LASC) and esophageal and lung tumors studied (Additional file 1: Figure S1). Examination of TrkB-T1 mRNA showed that, overall, the median levels of this transcript were similar in control and LASC samples, though there were a small number of tumors with extremely high levels of TrkB-T1 RNA (Fig. 1). OSSCs and, curiously, non-head and neck SCCs, such as LUSC and esophageal SCC (ESSC) showed the same pattern. Samples with TrkB-T1 mRNA levels above the mean tumor level were defined as high TrkB-T1 expressers. High TrkB-T1 expressers comprised 30% of tumors for LASC (vs. 0% of normal controls), 20% of tumors for OSCC (vs 21% of normal controls), 35% for LUSC (vs. 0% of normal controls), and 36% for ESSC (vs. 0% for normal controls). For adenocarcinomas, the fractions of tumors with TrkB-T1 expression over the average were 13% for LUAD (vs. 24% of normal controls) and 10% for ESAD (vs. 54% of normal controls) (Additional file 2: Table S1).

### High expression levels of TrkB-T1 RNA in LASC, OSCC, ESLC, LUSC correlate with gene signatures associated with neural tissue

**Fig. 2:**
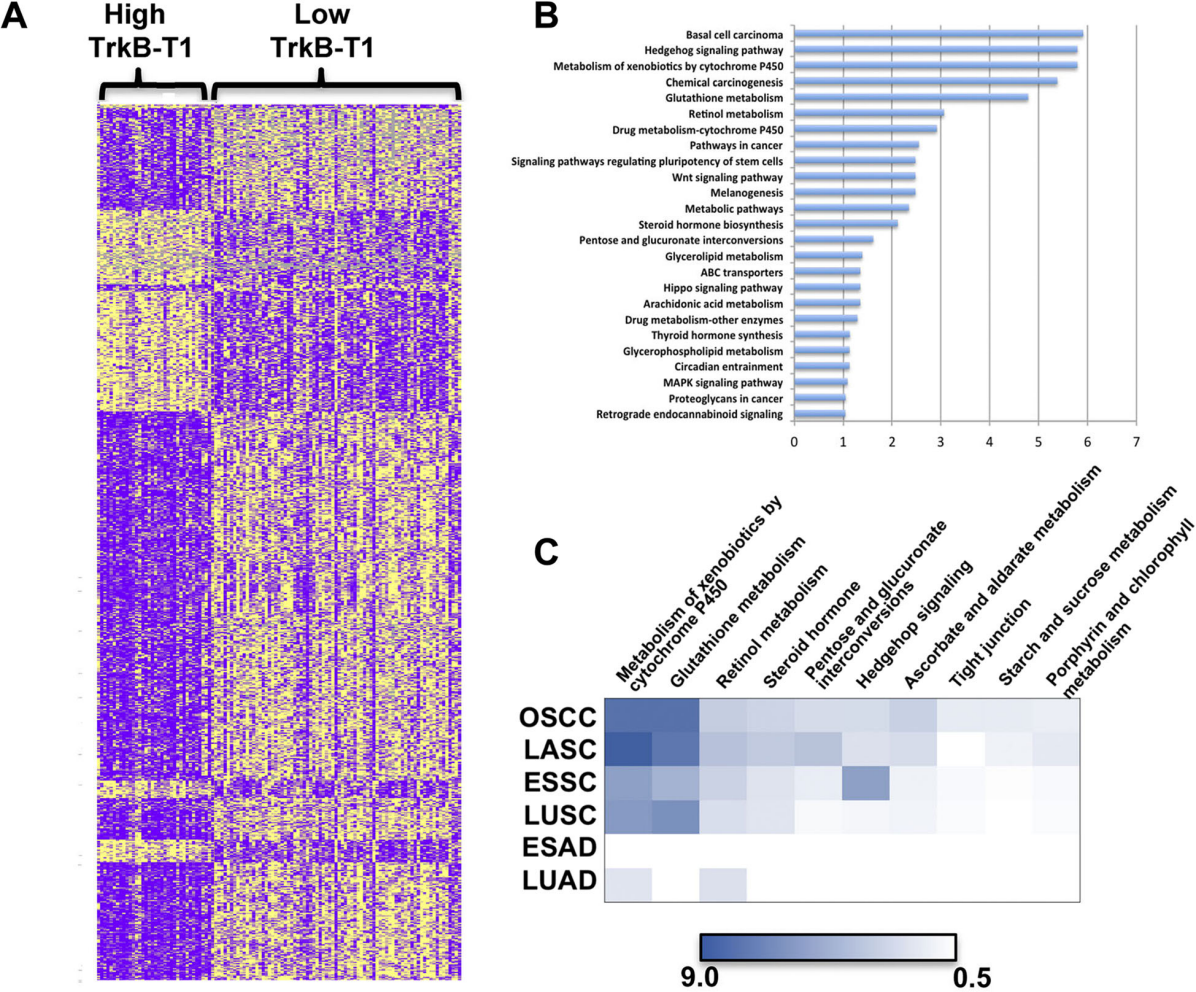
**a** Heat Map of RNA levels reveals DE genes in high versus low TrkB-T1 mRNA LASCs. **b** KEGG pathways that are elevated in high TrkB-T1 LASC samples from the TCGA database. Shown is negative log base 10 of the adjusted *P* value. **c** The heat map provides a summary of KEGG pathways enriched in TrkB-T1 high expressers cancers. All identified with the ENRICHR program. Shown is negative log base 10 of the adjusted P value

Gene signatures representing DE genes between the high and low TrkB-T1 expressers were identified for LASC (Fig. 2a) and OSCC. We note that a preponderance of genes more highly expressed in the TrkB-T1 high expressers were associated with neural tissue (Additional file 3: Table S2). Enrichr was used to identify transcriptomes of tissue samples in the GTEx Project of normal tissue transcriptomes, which showed expression profile differences similar to that of the high TrkB-T1 expressers [40, 49]. All of the top ten matches in the GTEx database were brain tissue samples for LASCs and OSCCs. When this analysis was done for high TrkB-T1 RNA expresser LUSCs, the similarity to brain tissue was seen, and for ESSC the top nine out of ten matches were brain tissue samples. In contrast, the above signature of TrkB-T1 high versus low expressers was not similarly linked to brain-like gene expression in adenocarcinoma. Eight out of ten transcriptome matches for the TrkB-T1 RNA high expresser LUADs were lung tissue samples, and for ESAD all ten were esophagus or vagina.

### Molecular pathways associated with TrkB-T1 mRNA enrichment

Enrichr was used to identify the biological processes associated with TrkB-T1 enrichment by probing the Kyoto Encyclopedia of Genes and Genomes (KEGG) pathways. A comparison of the DE genes in LASC TrkB-T1 high versus low expressers revealed a number of pathways enriched among the KEGG pathways as shown in Fig. 2b. Remarkably, when gene set analysis was done with a second head and neck SCC type, OSCC, from the TCGA database, a very similar group of pathways was shown to be overrepresented among the enriched genes in TrkB-T1 high expresser OSCCs (Fig. 2c, Additional file 4: Figure S2).

The next step was to determine if transcriptome patterns associated with high TrkB expression indicated similar pathways were enriched in tumor sites distinct from the head and neck. Again, the same pathways were seen to be enriched in high TrkB-T1 expressers versus low expresser ESSCs and LUSCs (Fig. 2c and Additional file 5: Figure S3). A second gene set analysis tool was used to verify common differences in biological pathways seen in high versus low TrkB-T1 expressers in SCCs. Gene set enrichment analysis (GSEA) uses a different approach and includes all changes in gene expression. KEGG pathways identified as differentially represented were largely the same in head and neck SCC (made up of LASC and OSCC combined), LUSC, and ESCC (Additional file 6: Table S3). Importantly, Enrichr analysis of ESAD showed no enriched KEGG pathways in the TrkB-T1 high expressers. Enrichr analysis of LUAD produced a group of pathways, including metabolism by CYP450, that was enriched in the high TrkB-T1 RNA expressers. That and the retinol metabolism pathway were included among pathways enriched in TrkB-T1 SCC high expressers, though the statistical significance of the enrichment was much weaker. Analysis by GSEA provided no statistically significant enriched pathways (Additional file 6: Table S3).

**Fig. 3:**
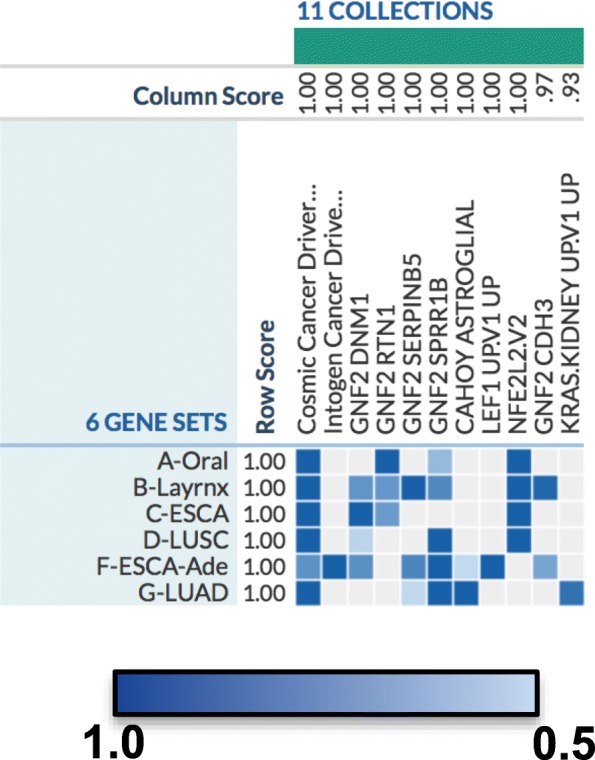
Heat Map Molecular Signal Database Oncology pathways enriched in TrkB-T1 high expresser tumors identified by KnowEnG Score. A score of 1 indicates a high association between the TrkT-T1 high expresser gene sets and the public gene sets listed; a score of 0.5 indicates a weak association

A third analysis using an alternative gene set analysis tool, KnowENG, was done to search the cancer pathway sets of the Molecular Signature Database (MSigDB). This analysis revealed that the Nfe2l2 pathway involved in cellular response to oxidizing insults was enriched most highly in all 4 high TrkB-T1 expresser SCCs tested, LASC, OSCC, ESSC and LUSC, and not in the high TrkB-T1 adenocarcinomas [41](Fig. 3).

All these DE analyses were based on TGCA datasets done with RNAseq analysis. To corroborate this approach, an independent RNA expression dataset from a different OSCC database with gene expression measured by a different approach, DNA microarray hybridization, was used (GSE30784) [50]. Results of this analysis revealed most of the same pathways were enriched in the TrkB-T1 high expressers (Additional file 5: Figure S3).

### Global protein analysis

**Fig. 4:**
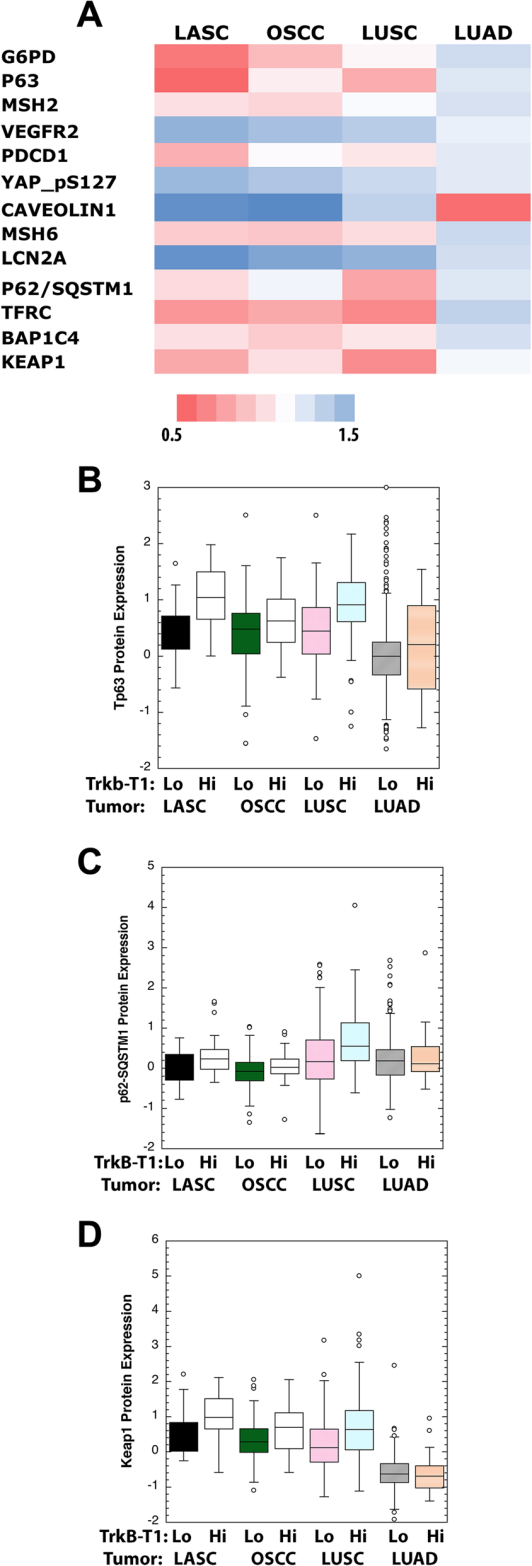
Reverse Phase protein and phosphoprotein analysis by the TCPA reveals differences in levels of Tp63, p62/SQSTM1, Keap1 and other proteins in TrkB-T1 high expresser LASCs and other SCCS. **a** Heat map reveals relative log2 protein levels that were differentially abundant based on univariate test at FDR < 0.10 for LASC. Levels in other tumor types are shown. Geometric means are shown. **b** Box plots show differential expression of Tp63 in high TrkB-T1 expresser LASCs vs. low expressers *p* < 0.00339, then for OSCC, *p* < 0.0509, LUSC, *p* < 0.0001 and adenocarcinoma, LUAD, *p* < 0.150 determined by Wilcoxon test. Shown are arithmetic means of values. **c** Similar to B except p62/SQTM1 was measured with differences shown for LASC at *p* < 0.0184, OSCC *p* < 0.0299, LUSC p < 0.0001 and LUAD *p* < 0.986. **d** Similar to B except Keap1 protein was measured with differences shown for LASC at p < 0.00339, OSCC *p* < 0.0410, LUSC p < 0.0001 and LUAD *p* < 0.350

Global protein measurement of TCGA samples by the TCPA reverse-phase protein array analysis [39] revealed 13 of 237 proteins tested showed small but consistent differences in levels at FDR < 0.1 in TrkB-T1 mRNA high expresser LASCs versus low expressers. Examination of levels of these 13 proteins in OSCCs and LUSCs showed similar differences in TrkB-T1 mRNA high expressers versus low expressers for G6pd, Tp63, Msh2, Vegfr2, Msh6, Lcn2a, p62/SQSTM1, Tfrc, Bap1c4, Keap1 (Fig. 4). These differences were not seen in the non-squamous cell carcinoma LUAD TrkB-T1 high expressers. TCPA analysis of ESSCs was not included because lower numbers of samples were assayed with fewer antibodies.

**Fig. 5:**
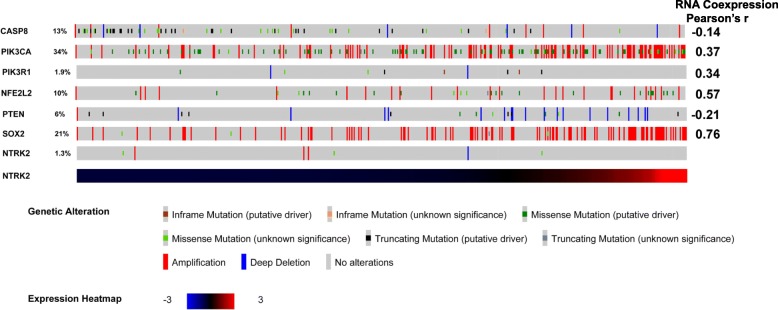
Copy number changes and mutations in known HNSCC oncogenes that may correlate with NTRK2 gene expression levels revealed using cBioPortal on TCGA HNSCCs. Samples are sorted by increasing NTRK2 mRNA level, as shown in the bottom bar which is a schematic of NTKK2 mRNA levels in each sample. The percentage on the left is frequency of mutations and amplifications over all for that gene. Shown on the right is the correlation between TrkB mRNA level and the level of the named mRNAs in that row

Some gene and gene expression changes associated with TrkB-T1 high level expression. cBioPortal was used to examine genes whose mutation, amplification and mRNA level are associated with head and neck SCC and are suspect drivers of SCC at that site [6]. This list of 20 genes includes CDKN2A, FAT1, TP53, CASP8, AJUBA, PIK3CA, NOTCH1, SOX2, and 12 others (Additional file 7: Figure S4). RNA levels of Sox2, transcriptional regulator in stem cells linked to the Sonic hedgehog pathway [51, 52] and Nfe2l2, a transcriptional regulator of Nfe2l2 pathway genes [53], correlated well with TrkB-T1 mRNA levels (Fig. 5). PIKC3 gene copy number correlated weakly with TrkB-T1 RNA at r = 0.3 and r = 0.47 in LASC and OSCC cells respectively. Pikc3a is a key component of the AKT pathway which in turn has been shown to be regulated by TrkB proteins [17, 18]. Logistic regression revealed that, in LASC, activating mutations of the PIK3CA gene were associated with high TrkB-T1 expression (odds ratio 0.720; 95% confidence interval, 0.577 to 0.899; *p* = 0.0037).

High level expression of TrkB-T1 is associated with poor survival of LASC but not LUSC.

**Fig. 6:**
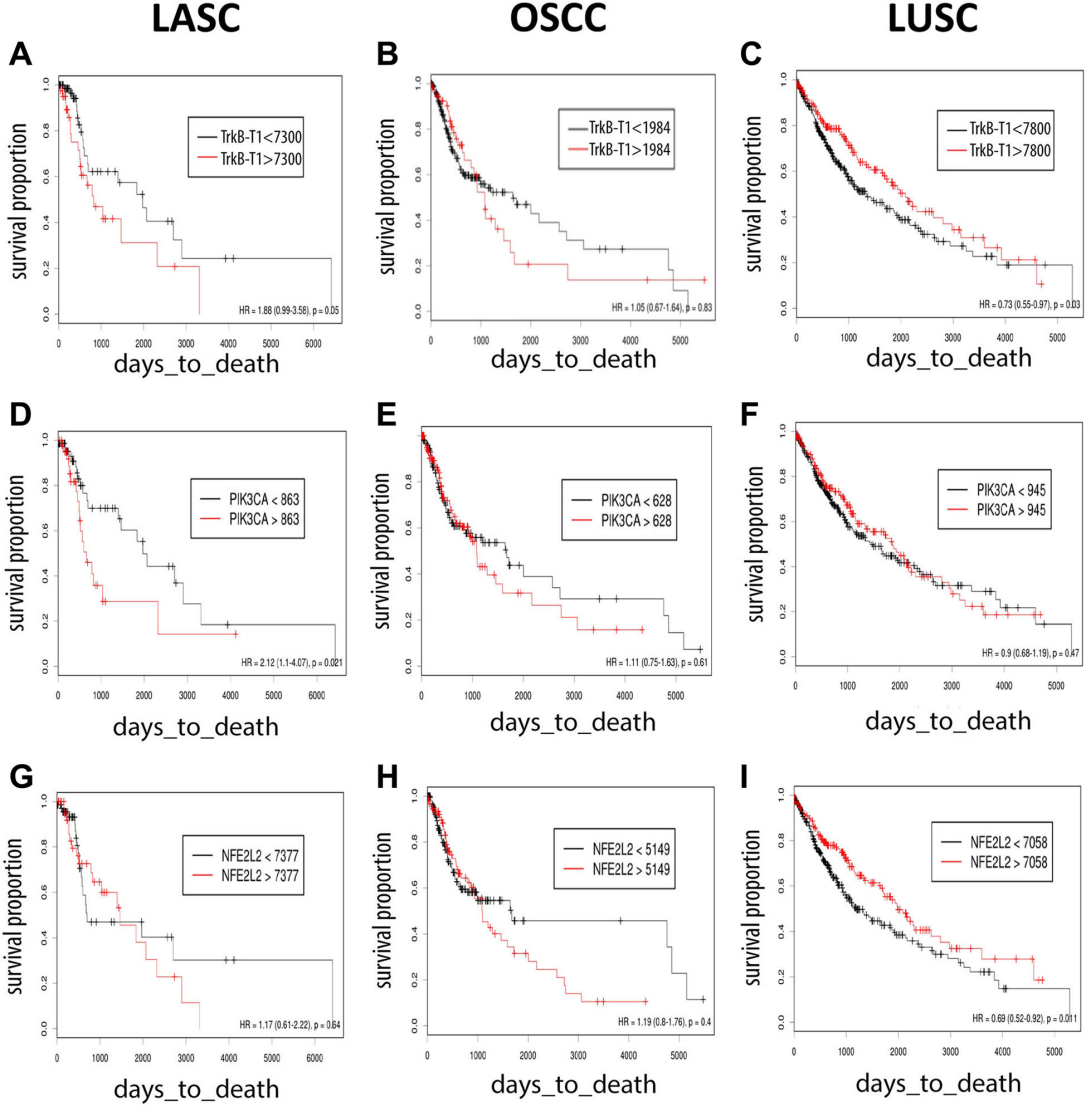
Outcomes as overall survival for **a**. NTRK2, **d**. PIK3CA and **g**. NFE2L2, high expresser LASCs; and **b** NTRK2, **e**. PIK3CA, **h**. NFE2L2 high expresser OSCCs; and **c**. NTRK2, **f**. PIK3CA and **i**. NFE2L2 high expressers LUSCs

We initially examined LASC patient samples because they showed a large number of tumors with very high levels of TrkB-T1 mRNA, ensuring sufficient number to do survival analysis. An examination of patient outcomes revealed that high expressers of TrkB-T1 had reduced overall survival (*p* < 0.04, Fig. 6a). A similar examination of OSCC revealed no differential survival for TrkB-T1 high expressers (Fig. 6b). In the first 1000 days, high levels of TrkB-T1 mRNA trended toward predicting better outcomes in that time period but not after that. An examination of a second dataset of subjects with adequate subject numbers through the first 1000 days saw the same thing (GSE65858) [54]. In the TCGA data set more of the high TrkB-T1 expresser tumors were advanced stage tumors (Additional file 2: Table S1).

An analysis of effects on survival was done in LUSC to test if extreme high TrkB had a negative effect on outcomes in this tumor subtype, as TrkB protein levels are thought to be a negative predictor in a wide range of cancer types [10–16, 36]. This was not the case for the high TrkB-T1 RNA expressers, which showed increased overall survival (Fig. 6C). An examination of two additional LUSC datasets revealed, in one, a similar pattern with TrkB high expressers showing better outcomes (GSE457) [55] while the other, with only 56 patients, showed no difference (GSE17710) [56]. In additional studies that looked at overall survival, there was a modestly lower level of TrkB-T1 RNA in subjects who had died by 1, 3, and 5 years after diagnosis again suggesting TrkB-T1 levels directly correlated with increased survival times for LUSC patients [55, 57].

PIK3CA copy number and Nfe2l2 RNA levels correlate with overall survival in LASC. PIK3CA and Nfe2l2 are genes whose copy number and or expression correlate, though weakly with TrkB-T1 mRNA levels in SCCS. For LASC, an examination of outcomes revealed patients whose tumors expressed higher level of PIK3CA, or had higher copy numbers of this gene, had worse outcomes on average (Fig. 6d). The same was true in OSCCs (Fig. 6e). Surprisingly, that was not the case for LUSC. LUSC tumors that were enriched for PIK3CA gene copy number trended toward showing favorable outcomes (Fig. 6f). This differential was duplicated in an examination of two separate studies of patients with LUSC, which showed 20–50% lower levels of PIK3CA gene expression among patients who were dead at 1, 3, and 5 years after cancer [55, 57]. Similarly, Nfe2l2 transcription factor and the gene products associated with this antioxidant pathway have been shown to be elevated in head and neck SCC and LUAD tumors of patients who show poor outcomes, while for LUSCs this was not the case [58, 59]. The TCGA dataset showed unclear effects of Nfe2l2 mRNA enrichment on LASC and OSCC outcomes, but did see a survival benefit to enrichment of the mRNA among LUSC patients (Fig. 6g-i).

## Discussion

The original intents of this study were to use the accuracy of RNA measurement to characterize SCCs based on NTRK2 gene expression and to develop a reproducible method to identify aggressive tumors. The observation that TrkB-FL makes up only a tiny percentage of total NRTK2 gene mRNA put the focus on TrkB-T1 mRNA (Additional file 1: Figure S1). TrkB protein analysis in many studies has indicated higher levels of TrkB protein are found in a large subset of tumor types, but has not allowed a thorough description of the differences in high versus low TrkB expressing tumors [8, 10, 16, 17]. The work described here showed that TrkB-T1 RNA, which is at high levels and can be measured accurately, can be used to subclassify SCC tumors of different organs as a group which turn out to have some shared properties.

The TrkB-T1 high expresser tumors fall mainly into two of four head and neck SCC subtypes described earlier, atypical and classical [4, 5]. They showed increased expression of.

NFE2l2, SOX2, and PIK3CA associated with both those subclasses of head and neck SCC [4]. Compared to tumors with low-level expression of TrkB-T1, the TrkB-T1 high expressing tumors showed increased levels of neural specific mRNAs (Additional file 3: Table S2). These high TrkB-T1 SCCs would likely be a subset of the neuroendocrine-like tumors or C4 classification of Chen et al. from their TCGA pan-cancer analysis, though they are distinct in that only SCCs are included and they all express TrkB-T1 mRNA at high level [60]. In that TrkB plays a role in neuronal cell behavior in development, it may contribute to regulation of neural-specific gene expression in these tumors. TrkB-T1 expression showed a strong correlation in OSCC, LASC, ESSC, and LUSC tumors groups in this study with the mRNA for the Sox2 neural developmental transcription factor [51, 52, 61]. Other candidates to control the high TrkB-T1 SCC mRNA levels seen in these tumors include Tcfap2a, a transcription factor involved in neural crest formation and/or function [62]. The Tcfap2a transcription factor binding site sequence was overrepresented in the promoters of DE genes in high TrkB-T1 mRNA expressers versus low expressers in all 4 SCCs studied [32, 44].

SCCs, whether laryngeal, oral, lung, or esophageal, that expressed high levels of TrkB-T1 mRNA also showed enrichment of the same KEGG pathways identified by gene set analysis compared to their low TrkB-T1 counterparts. (Fig. 2c). This duplication of results with high TrkB SCCs at different body sites solidifies the association of these pathways with TrkB-T1 mRNA expression and these SCCs as a distinct entity. The enriched pathways included hedgehog, long associated with Basal Cell Carcinoma and normal development [63]. This pathway could play a role in tumor formation and progression in SCCs [64, 65]. Sox2 may serve as a link between TrkB-T1 RNA levels and the hedgehog pathway. Sox2 has been shown to play a role in the function of hedgehog signaling [51, 66–68]. Specific proteins of the pathway and Sox2 work together to determine cell fate. The mechanism for co-expression of TrkB-T1 and Sox2 in these SCCs is unclear [61]. Analysis of 237 proteins as part of the Cancer Proteome Atlas revealed Tp63 which stimulates expression of sonic hedgehog pathway genes in mammary cancer stem cells was enriched in high TrkB-T1 expresser SCCs (Fig. 4) [69].

A second pathway, retinol metabolism, which includes differentially expressed enzymes, some of which can deactivate retinols, suggests the idea that TrkB-T1 high expresser SCCs would be resistant to retinoids [70], a family of drugs once tested for their curative effects on OSCC, but found to be ineffective overall [71, 72]. Gene set analysis among disease- and drug-related pathways in the MSigDB revealed that a large number of the 450 genes that comprise the Nfe2l2 pathway were enriched in the TrkB-T1 high expressers of each SCC. This occurred to a much lesser degree in the two adenocarcinoma groups (Additional file 4: Figure S2). Nfe2l2 induces transcription of genes of the antioxidant and detoxification pathways [53]. These include glutathione metabolism and xenobiotic/drug metabolism by cyp450, two KEGG pathways associated with high TrkB-T1 mRNA levels in SCC. In non-SCC breast cancer, TrkB kinase activity has been shown to reduce levels of the Keap inhibitor, which increases Nfe2l2 directed transcription [73]. In addition BDNF has been shown to induce Nfe2l2 mRNA in astrocytes via TrkB-T1 activation [74]. Keap1 protein was enriched in high TrkB expressing LASC, OSCC, and LUSC, as was p62/SQSTM1 (Fig. 4). Keap1 is an inhibitor of the Nfe2l2 pathway, while p62/SQSTM1 is an activator [53, 75]. In that many Nfe2l2 targets are enriched in TrkB-T1 high expresser SCCs one might speculate that p62/SQSTM1 is more active in those tumors.

While the correlation between TrkB-T1 mRNA level and PIK3CA copy number was marginal in the SCCs (Additional file 8: Table S4), the functional interaction of these genes in these tumors was further supported by the increase in mutagenic PIK3CA activation in high TrkB expressers in LASC for example (Fig. 6). We note TrkB-FL kinase is known to work via activation of PI3K and downstream AKT [17, 18]. Recent work showed TrkB-T1 can activate ERK and AKT signaling both of which are downstream of Pi3K [76]. These and other findings allow speculation that PI3K activation and increased transcription of TrkB-FL and TrkB-T1 are mutually stimulatory [9, 77, 78]. This study provides some of the first evidence Sonic Hedgehog and Retinol metabolism are related to NTRK gene expression in SCC tumors, and some of the first for the Nfe2l22 pathway. However, a limitation of the study is that it is not clear if TrkB-T1 or, for that matter, TrkB-FL protein contribute to specific patterns of gene expression seen in the TrkB-T1 high expressers.

## Conclusions

The observation that high TrkB-T1 LASC patients show poor outcomes, while the opposite is true in high TrkB-T1 LUSCs creates a question (Fig. 6a and c). If TrkB-T1 mRNA enrichment and the set of pathways associated with this enrichment in the analysis are relevant to this TrkB-high SCC tumor subtype then why do TrkB-T1 high expressers still have organ site-specific properties in regard to patient outcomes? Importantly, enrichment of TrkB-T1 mRNA is correlated to varying degrees with enrichment of Nfe2l2 pathway, Pik3CA of the PI3K pathway and hedgehog pathways and Sox2. These are pathways and factors known to have SCC site-specific effects on patient outcomes and on resistance to radio- and chemotherapy in at least some tumor types, [58, 79–82]. Pik3ca enrichment in head and neck SCC predicts poorer outcomes [80, 81] while amplified PIKC3A in LUSC may have the opposite effect [83](Fig. 6). Likewise, enrichment of factors linked to activated Nfe2l2 are associated with poor survival of head and neck SCC and LUAD, but that is not the case for LUSC and other cancers [58, 59, 82, 84, 85]. Finally, Sox2 has tumor-specific associations with patient outcomes similar to TrkB: negative in LASC, mixed for head and neck SCC overall, and positive in LUSC [79, 86, 87]. So, while SCCs with high levels of TrkB–T1 mRNA may make up a subtype of SCC, where the same suite of pathways is enriched, the tumor behavior continues to depend on how the cell responds to activation of those pathways. This can be due to differences in background gene expression in the high TrkB-T1 SCCs at different sites or can be due to external differences associated with that tumor site, such as the standard treatment for that tumor type.

## Additional files

Additional file 1: **Figure S1.** Relative level and proportion of NTRK2 RNA that is TrkB-T1 based on RSEM counts for exon 16 of NTRK2, TrkB-FL, exon 23 and 24 of NTRK2, and TrkB-SHC, exon 19 of NTRK2. (PNG 237 kb)

Additional file 2: **Table S1.** Clinical characteristics of cancer patients with high and low TrkB-T1 mRNA expression levels. (XLSX 18 kb)

Additional file 3: **Table S2.** Of the 194 genes whose expression was enriched >3x in high TrkB-T1 mRNA expresser OSCCs, 73 are also highly enriched in normal neurological tissue. (XLSX 14 kb)

Additional file 4: **Figure S2.** KEGG pathways that are elevated in high expresser TrkB-T1 OSCC samples from the TCGA database. Negative log10 of the adjusted *P* value. All identified with ENRICHR program. Samples were divided into two groups above and below the mean TrkB-T1 mRNA level with the exception of ESAD where only the top 30 samples in each group were included to determine the DE gene list. Shown are values for A. OSSC, B. ESSC, C LUSC and D LUAD. ESAD showed no enriched pathways. (PDF 335 kb)

Additional file 5: **Figure S3.** KEGG pathways that are enriched in TrkB-T1 high expressers in an earlier study of OSCC gene expression, (GSE30784) [50] measured by DNA hybridzation arrays determined using Enrichr. 167 patients total with 17 high TRKB expressers above the mean. Shown is negative log base 10 of the adjusted *p* value. TrkB-T1 levels are average of 3 probe sets of the specific for TrkB-T1, 221795_at, 221796_at and 214680_at of the Affymetrix Human Genome U133 Plus 2.0 Array. (PDF 65 kb)

Additional file 6: **Table S3.** Gene Set Enrichment Analysis of pathway enrichment in TrkB-T1 high expresser SCCs. (XLS 19 kb)

Additional file 7: **Figure S4.** Composite view of mutation and amplification events for genes previously identified as potential drivers of HNSCC using cBioPortal with addition of NTRK2. Samples are sorted by increasing NTRK2 mRNA level, as shown in the bottom bar which is a schematic of NTKK2 mRNA levels in each sample. The percentage on the left is frequency of mutations and amplifications. Shown on the right is the correlation between TrkB mRNA level and the level of the named mRNAs. (PNG 647 kb)

Additional file 8: **Table S4.** Correlation of TrkB-T1 mRNA level and oncogene mRNA and or gene copy number for TCGA datasets. (DOCX 12 kb)

## Data Availability

The datasets used and/or analyzed during the current include gene expression (RNAseq V2), CNA copy number amplification and clinical data originally curated by TCGA Research Network available from the Broad Institute TCGA GDAC Firehose repository via Firebrowse (https://gdac.broadinstitute.org/). The Cancer Proteome Atlas (TCPA) reverse-phase protein array analysis data (V4.2, 2018/07/18) are available at https://www.tcpaportal.org/tcpa/download.html.

## Abbreviations

DE: Differentially expressed
ESAD: Esophageal adenocarcinoma
ESSC: Esophageal squamous cell carcinoma
GSEA: Gene set enrichment analysis
KEGG: Kyoto Encyclopedia of Genes and Genomes
LASC: Laryngeal squamous cell carcinoma
LUAD: Lung adenocarcinoma
LUSC: Lung squamous cell carcinoma
NFE2L2: Nuclear factor (erythroid-derived 2)-like 2
OSCC: Oral squamous cell carcinoma
PIK3CA: Phosphatidylinositol-4,5-bisphosphate 3-kinase catalytic subunit alpha
SOX2: SRY-Box 2
TCGA: The Cancer Genome Atlas
TRKB: Tropomysin-related receptor kinase B
