# Supplementary figures and images for: A subtype of oral, laryngeal, esophageal, and lung, squamous cell carcinoma with high levels of TrkB-T1 neurotrophin receptor mRNA
Source: BMC Cancer. 2019 Jun 20;19:607. doi: 10.1186/s12885-019-5789-8 (PMC6587277; doi:10.1186/s12885-019-5789-8)

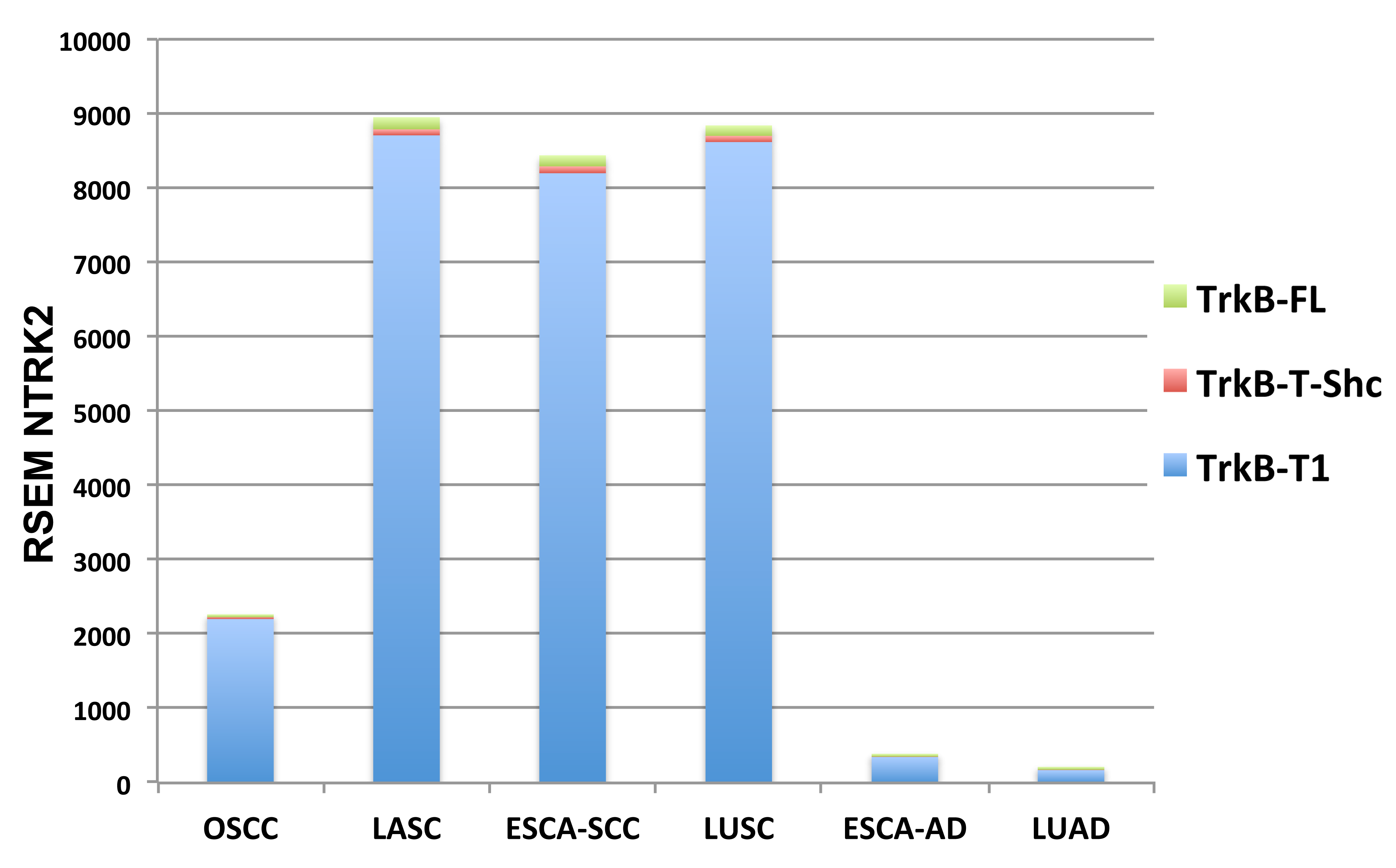

Supplement: Supplementary file 1 — Figure S1. Relative level and proportion of NTRK2 RNA that is TrkB-T1 based on RSEM counts for exon 16 of NTRK2, TrkB-FL, exon 23 and 24 of NTRK2, and TrkB-SHC, exon 19 of NTRK2. (PNG 237 kb) [file 12885_2019_5789_MOESM1_ESM.png]

## A. OSCC

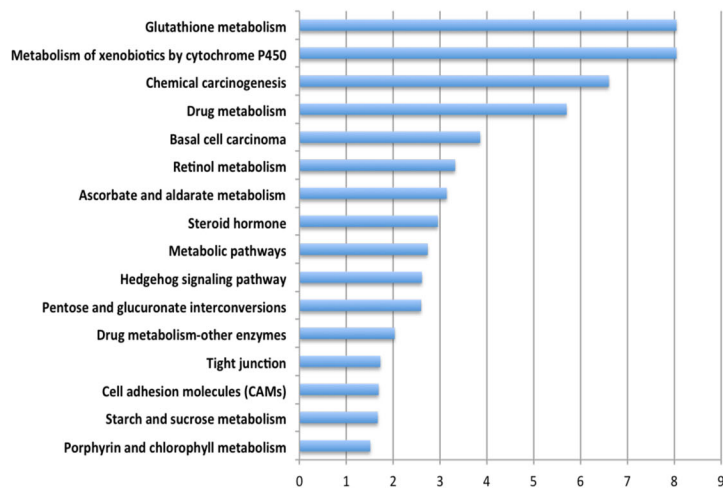

## B. ESSC

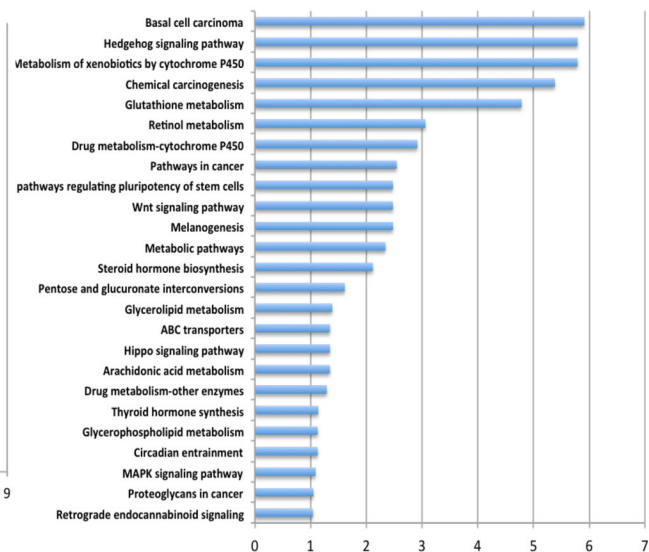

## C. LUSC

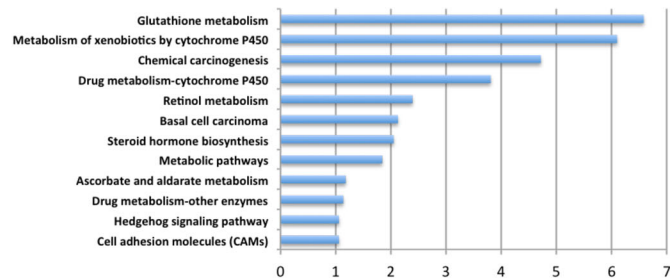

## D. LUAD

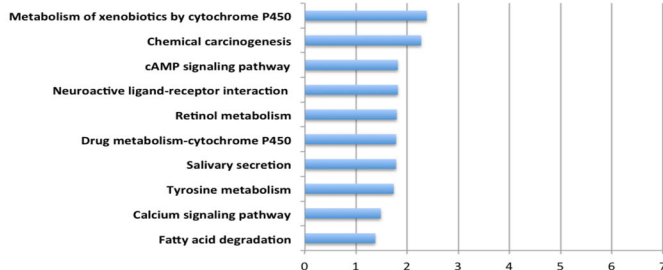

Supplement: Supplementary file 4 — Figure S2. KEGG pathways that are elevated in high expresser TrkB-T1 OSCC samples from the TCGA database. Negative log10 of the adjusted P value. All identified with ENRICHR program. Samples were divided into two groups above and below the mean TrkB-T1 mRNA level with the exception of ESAD where only the top 30 samples in each group were included to determine the DE gene list. Shown are values for A. OSSC, B. ESSC, C LUSC and D LUAD. ESAD showed no enriched pathways. (PDF 335 kb) [file 12885_2019_5789_MOESM4_ESM.pdf]

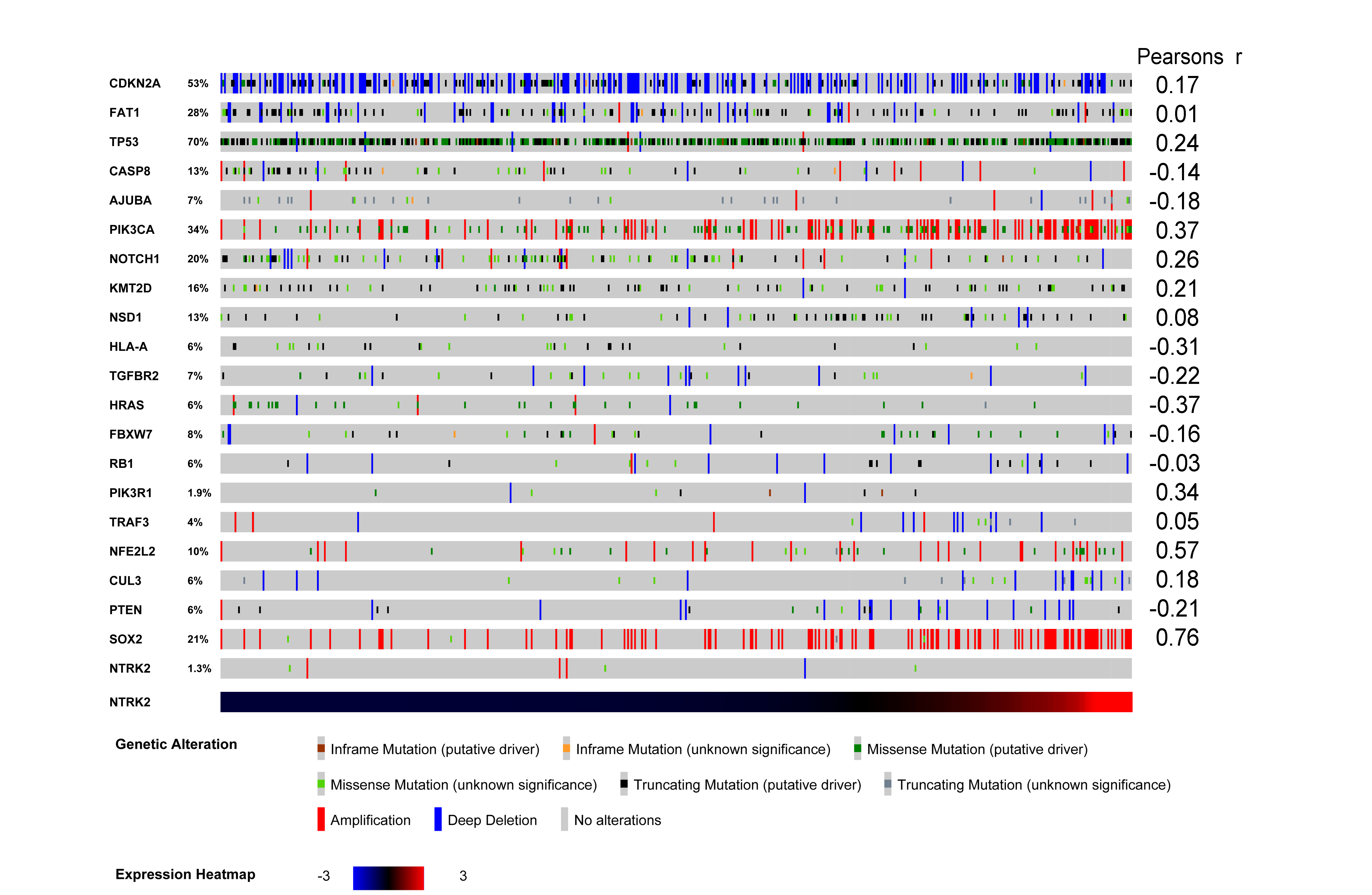

Supplement: Supplementary file 7 — Figure S4. Composite view of mutation and amplification events for genes previously identified as potential drivers of HNSCC using cBioPortal with addition of NTRK2. Samples are sorted by increasing NTRK2 mRNA level, as shown in the bottom bar which is a schematic of NTKK2 mRNA levels in each sample. The percentage on the left is frequency of mutations and amplifications. Shown on the right is the correlation between TrkB mRNA level and the level of the named mRNAs. (PNG 647 kb) [file 12885_2019_5789_MOESM7_ESM.png]
