# Supplementary material for: A subtype of oral, laryngeal, esophageal, and lung, squamous cell carcinoma with high levels of TrkB-T1 neurotrophin receptor mRNA
Source: BMC Cancer. 2019 Jun 20;19:607. doi: 10.1186/s12885-019-5789-8 (PMC6587277; doi:10.1186/s12885-019-5789-8)
Supplement: Supplementary file 5 — Figure S3. KEGG pathways that are enriched in TrkB-T1 high expressers in an earlier study of OSCC gene expression, (GSE30784) [50] measured by DNA hybridzation arrays determined using Enrichr. 167 patients total with 17 high TRKB expressers above the mean. Shown is negative log base 10 of the adjusted p value. TrkB-T1 levels are average of 3 probe sets of the specific for TrkB-T1, 221795_at, 221796_at and 214680_at of the Affymetrix Human Genome U133 Plus 2.0 Array. (PDF 65 kb) [file 12885_2019_5789_MOESM5_ESM.pdf]

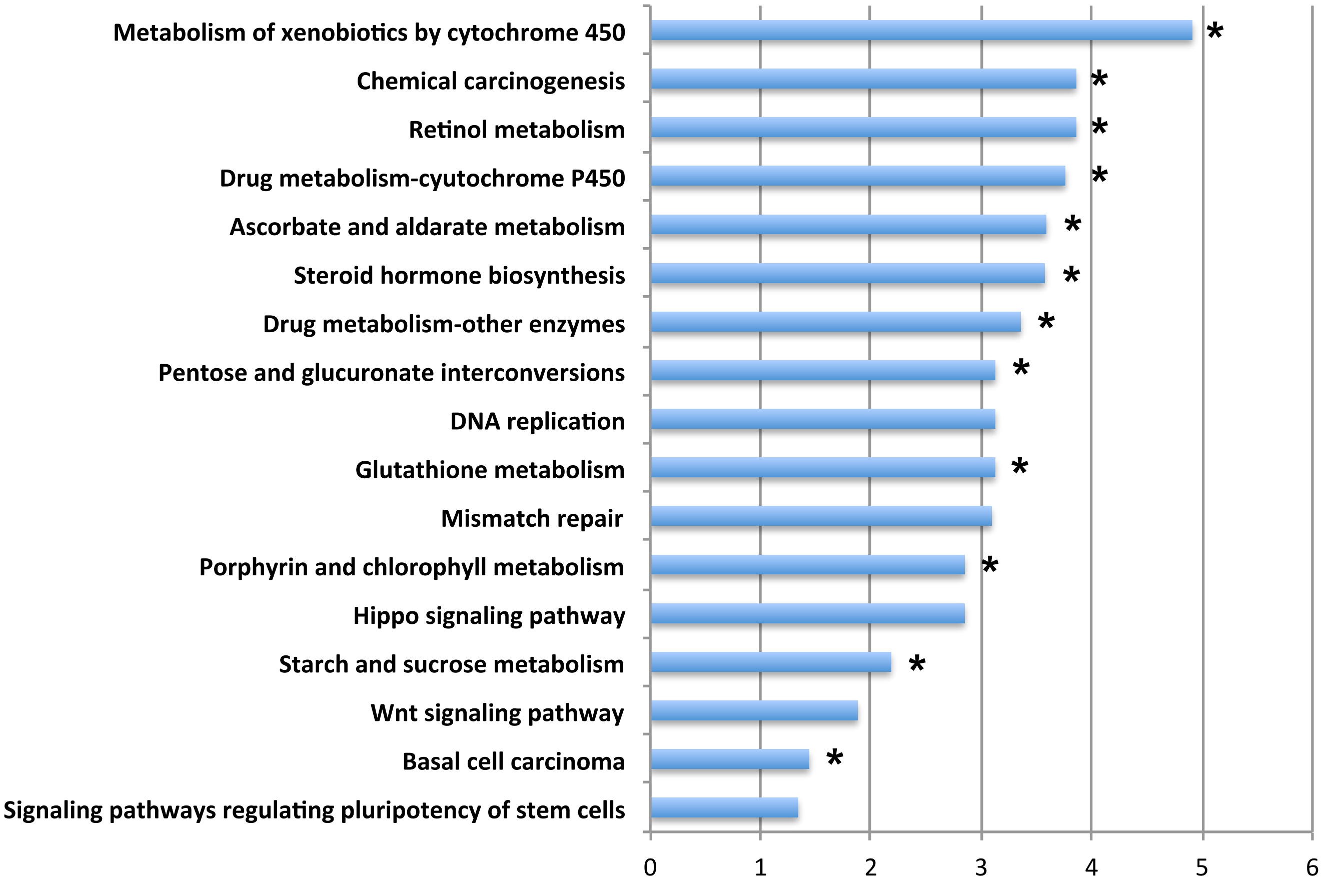
Metabolism of xenobiotics by cytochrome 450
Chemical carcinogenesis
Retinol metabolism
Drug metabolism-cyutochrome P450
Ascorbate and aldarate metabolism
Steroid hormone biosynthesis
Drug metabolism-other enzymes
Pentose and glucuronate interconversions
DNA replication
Glutathione metabolism
Mismatch repair
Porphyrin and chlorophyll metabolism
Hippo signaling pathway
Starch and sucrose metabolism
Wnt signaling pathway
Basal cell carcinoma
Signaling pathways regulating pluripotency of stem cells
*
0
1
2
3
4
5
6
