# Supplementary material for: A subtype of oral, laryngeal, esophageal, and lung, squamous cell carcinoma with high levels of TrkB-T1 neurotrophin receptor mRNA
Source: BMC Cancer. 2019 Jun 20;19:607. doi: 10.1186/s12885-019-5789-8 (PMC6587277; doi:10.1186/s12885-019-5789-8)
Supplement: Supplementary file 8 — Table S4. Correlation of TrkB-T1 mRNA level and oncogene mRNA and or gene copy number for TCGA datasets. (DOCX 12 kb) [file 12885_2019_5789_MOESM8_ESM.docx]

| **Column1** | **Nfe2l2 mRNA** | **Pik3ca mRNA** | **PIK3CA CNA** | **Sox2 mRNA** |  |
| --- | --- | --- | --- | --- | --- |
| OSSC | 0.536 | 0.251 | 0.309 | 0.648 |  |
| LASC | 0.604 | 0.421 | 0.469 | 0.863 |  |
| ESSC | 0.533 | 0.406 | 0.451 | 0.782 |  |
| LUSC | 0.665 | 0.596 | 0.553 | 0.707 |  |
| ESAD | 0.370 | 0.119 | -0.0354 | 0.201 |  |
| LUAD | 0.418 | 0.169 | 0.0863 | -0.0047 |  |
|  |  |  |  |  |  |
| Supp. Table 4 Correlation of TrkB-T1 mRNA level and oncogene mRNA and/or | | | | | |
| gene copy number for TCGA datasets. Shown is Pearson correlation. | | | | |  |
